# The dimensional structure of the functional abilities in cases of long-term sickness absence

**DOI:** 10.1186/1471-2458-11-99

**Published:** 2011-02-14

**Authors:** Jake PJ Broersen, Henny PG Mulders, Antonius JM Schellart, Allard J van der Beek

**Affiliations:** 1 Research Centre for Insurance Medicine, collaboration between AMC-UMCG-UWV-VUmc, Amsterdam, the Netherlands; 2 Department of Public and Occupational Health, EMGO Institute for Health and Care Research, VU University Medical Centre, Amsterdam, the Netherlands; 3 Knowledge Centre of the Employee Insurance Authority, Amsterdam, the Netherlands

## Abstract

**Background:**

The health problems that working people suffer can affect their functional abilities and, consequently, can cause a mismatch between those abilities and the demands of the work, leading to sickness absence. A lasting decrease in functional abilities can lead to long-term sickness absence and work disability, with negative consequences for both the worker and the larger society. The objective of this study was to identify common disability characteristics among large groups of long-term sick-listed and disabled employees.

**Methods:**

As part of the disability benefit entitlement procedure in the Netherlands, an insurance physician assesses the functional abilities of the claimant in a standardised form, known as the List of Functional Abilities (LFA), which consists of six sections containing a total of 106 items. For the purposes of this study, we compiled data from 50,931 assessments. These data were used in an exploratory factor analyses, and the results were then used to construct scales. The stability of dimensional structure of the LFA and of the internal consistency of the scales was studied using data from 80,968 assessments carried out earlier, under a slightly different legislation.

**Results:**

Three separate factor analyses carried out on the functional abilities of five sections of the LFA resulted in 14 scale variables, and one extra scale variable was based on the items from the sixth section. The resulting scale variables showed Cronbach's Alphas ranging from 0.59 to 0.97, with the exception of one of 0.54. The dimensional structure of the LFA in the verification population differed in some aspects. The Cronbach's Alphas of the verification population ranged from 0.58 to 0.97, again with the exception of the same scale: Alpha = 0.49.

**Conclusion:**

The differences between the dimensional structures of the primary data and the earlier data we found in this study restrict the possibilities to generalise the results. The scales we constructed can be utilised to produce a compact description of the functional abilities of groups of claimants in the Netherlands. Moreover, the matching work demands can be used to identify jobs low on those demands as being the most accessible for the specific type of disabled employees, particularly severely disabled individuals.

## Background

Long-term sickness absence and work disability stem from a mismatch of the job demands on the one hand and the capacities of the employee on the other. The Organisation for Economic Co-operation and Development (OECD) studied the extent of the problem of long-term sickness and work disability in a number of OECD member states. This problem increased in a majority of those countries over the period from 1990 through 1999, although the increase in work disability slowed during the second half of the 1990s, and in a number of countries, the inflow into disability benefits even declined [1]. Nevertheless, public expenditures on disability benefits in 20 OECD member states amounted to a mean of 2.25% of the Gross Domestic Product (GDP) in 1999. Surveys conducted by Eurostat also looked at chronic illness and disability in the European Union: the 1996 estimate for the prevalence rate for chronic disease and disability (moderate and severe) in the working-age population was 14.5% [2,3]. In the Netherlands, the number of disability benefit recipients decreased in recent years [4], but more than 850,000 individuals out of a labour force of nearly ten million received a disability benefit in 2007 [5].

In the Netherlands, the remaining work capacity of a long-term sick-listed employee is assessed by matching the work demands of various jobs with the functional abilities of the sick-listed employee. An insurance physician (IP) assesses those functional abilities, which are then registered in a standardised format known as the List of Functional Abilities (LFA), which was partly based on the International Classification of Functioning, Disability and Health (ICF [6]). Subsequently, each functional ability is compared separately to the corresponding work demand in various jobs by a labour expert (LE). However, many of the functional abilities of employees are not statistically independent, nor are many of the demands within jobs. To simplify the description of those various employee and job characteristics, attempts can be made to summarise these characteristics in a restricted number of dimensions. On the job side, reducing the demands to a limited number of dimensions facilitates the clustering of jobs into job types [7-11]. The number of dimensions identified in these studies varied from two to nine, leading to four to 15 occupational categories. Schellart et al. [11], for example, distinguished two main dimensions: mental demands and physical demands. The study identified three job types mainly involving varying degrees of physical demands; two types involving mainly mental demands; and one type involving mixed types of demands.

In Norway, information on the functional abilities of sick-listed employees was registered using the Norwegian Function Assessment Scale (NFAS) [12,13]. The 39 items of the NFAS were summarized in seven scales, which reduce the amount of information to four physical dimensions of functional abilities and three mental dimensions. These seven scales may aid professionals in assessing the work capacity of employees.

In 2004, the European Union of Medicine in Assurance and Social Security (EUMASS) created a working group on ICF. This working group proposed a ICF core set for functional assessment in disability claims in European social security systems, based on consensus among 20 members of the working group from 11 countries [14]. The ICF core set was intended to represent the common denominator, to be applied in all assessments and to be supplemented by other categories according to national standards and legislation. The proposed ICF core set consists of 20 functional (dis-)abilities.

Identification of dimensions within the various functional abilities (or disabilities) of disability benefit claimants could potentially produce valuable insight into the common disability characteristics of large groups within the population of claimants, who are all long-term sick-listed employees. These common disability characteristics of a group of claimants could be a common cause for the problems in matching their functional abilities with the work demands in their former jobs, and most likely in various other jobs. Insight into these problems could enable us to identify obstacles and opportunities for return to work, and to select potentially suitable job alternatives for these groups of claimants. These large groups of claimants with common disability characteristics vary in the extent of their disability. The claimants with the most severe disabilities face the most significant hurdles in their return to work [15], and for them it is most important to identify low-demand jobs.

The purpose of the current study was to identify common disability characteristics of groups of sick-listed and disabled employees. By determining dimensions within the LFA, we will facilitate the development of a concise disability profile, which can be used to monitor trends in disability claims; to assess the determinants of dependency on a disability benefit; and to identify problems in matching the functional abilities of groups of sick-listed and disabled employees with the work demands in various jobs. As a result of this last application, jobs that are particularly low in specific demands can be identified, which are most likely to be very accessible jobs for those groups of employees with the corresponding disability (assuming the absence of other major impediments in those jobs). To achieve this, we aimed to determine the dimensions within the LFA, their reliability and the stability of these findings.

## Methods

In the Netherlands, the assessment of work disability is facilitated by using a (computer) system, called the Claim Assessment and Monitoring System, or CAMS [16]. Part of the system is the List of Functional Abilities (LFA), which is used to register the assessment of functional abilities by insurance physicians (IPs), to be matched to the work demands in various jobs by a labour expert (LE). The LFA consists of 106 items, more than two thirds of which are dichotomous indicating the presence or absence of a specific ability. Nearly one-third of the items are polytomous, with three through five ordinal scoring categories for the severity of the disability. The items of the LFA are categorised into six sections: I personal functioning (30 items), II social functioning (17 items), III adjusting to the physical environment (13 items), IV dynamic movements (31 items), V static posture (11 items), and VI working hours (4 items). The monitoring part of the CAMS enabled us to study the LFA data of claimants for a disability benefit in secondary data analyses. In the interest of protecting claimants' privacy, the data used in our study are not openly available for others.

The data used in the primary analysis of this study originated from LFA records of assessments made from October 2005 through September 2007. No LFA data were available for two specific types of claimants: the first type of claimant was able to return to work in their former job, according to the judgement of the IP; the second type was too seriously disabled, for example bedridden, and a search for suitable jobs was considered useless. For the exploratory factor analyses, we used the LFA data of 50,931 claimants.

The predecessor to the current social insurance law WIA was called the WAO. Under the WAO, the functional abilities of claimants were assessed in a comparable way, and also registered in LFA data files. LFA data from assessments were available from July 2003 through March 2005. No LFA data were available for the same two types of claimants as described in the previous paragraph. In this study, the WAO data were used to study the stability of the dimensional structure of the LFA and the internal consistency of the scales in an earlier time period and under slightly different legislation, and in a different population. The main difference between the two populations is the time interval from the start of the sick leave until the assessment by the IP: approximately one year under WAO and two years under WIA. Under WIA, a prolonged time interval until the assessment may lead to the recovery of some of the relatively healthy sick-listed employees in the second year. Therefore, after a two-year sickness absence period, a relatively unhealthy group remains. A difference in the composition of the population may lead to other interrelations between variables, and thus to differences in dimensional structure and scale characteristics. For the analysis on the data from the WAO legislation period we used the data of 80,968 claimants. Claimants with incomplete data were excluded from both the WIA and the WAO analyses.

To avoid that the differences between sections would dominate the results, we analysed the sections separately, except two combinations of related sections: I + II and IV + V.

Not all items on the LFA were eligible for the study of the structure within the LFA. In the analyses, 26 items were precluded, leaving 80 items. Most of the excluded items of the LFA were excluded for their heterogeneous meaning, i.e. these items did not refer to a specific (restriction in) capacity. An example of just such an excluded item was the final item of section VI regarding the working time: "There are other restrictions to the working hours, i.e. . . . ". The IP indicated the presence or absence of other restrictions (yes/no), and, if so, the IP had to describe the nature of the restriction (in free format).

**Figure 1:**
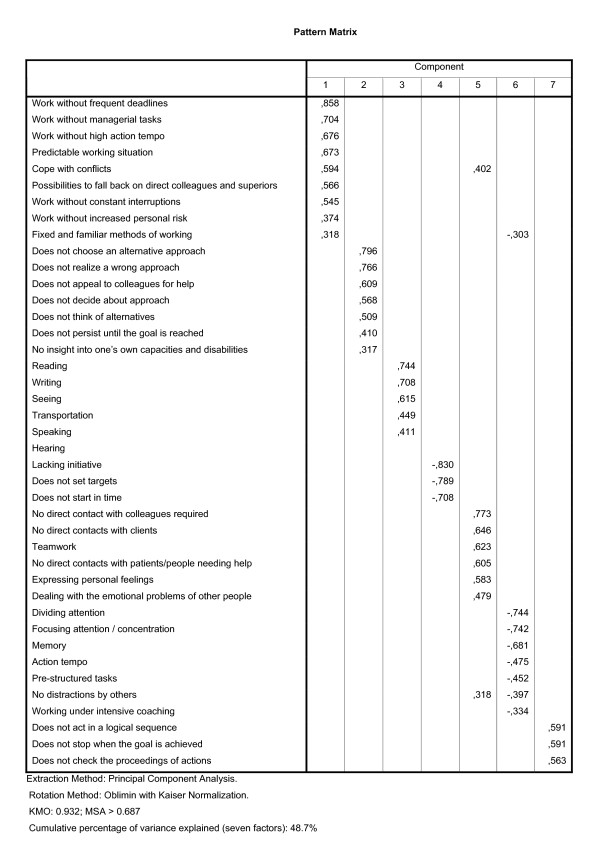
**Pattern matrix of the factor analysis on sections I and II of the LFA, using WIA data**. Section I includes 26 items related to personal functioning and section II includes 15 items on social functioning.

The preclusion of one item with a heterogeneous meaning of section VI reduced the number of items of section VI to three items, and we decided not to use factor analysis but only reliability analysis to study the relations between the three remaining items. As a result of the above-mentioned choices, 77 items were used in three factor analyses. The selected items for each analysis are described briefly in the figures 1 (section I and II), 2 (section III), and 3 (section IV and V).

The interrelations between items within sections of the LFA were analyzed using factor analysis of the SPSS package, opting for principal components analysis and oblimin rotation. In choosing the most suitable number of factors in the analyses on the WIA data, a minimum value of 1 of the so-called eigenvalues was used as a first criterion. Further decisions on the number of factors were based mainly on the plausibility of the resulting factors. As the items within a factor had more clear common characteristics, a factor was subjectively regarded as more plausible and nameable. The results of the factor analysis were used to construct scales. The items were assigned to the scales according to the highest coefficient in the pattern matrix, except under the following conditions: 1) the content shows a closer resemblance to the items of the alternative scale; 2) the addition of the item to the alternative scale should not weaken the statistical quality of this alternative scale, and preferably even strengthen it. The internal consistency of the scales was assessed by reliability analysis of the SPSS package. In many studies, Cronbach's Alphas of 0.70 or more are considered to be adequate. Instead of this usual value, we consider a value of the Cronbach's Alpha of 0.60 to be acceptable, because the items of the LFA were originally not selected to measure a common dimension. If the items of a list are not meant to measure a limited number of common dimensions, then the expected correlations between the items of scales within the list is lower, and consequently the Cronbach's Alphas will more rarely reach a level that is usual for scale construction. For the factor analyses on the WAO data we chose to use the same type of analysis as the initial analyses on the WIA data, with an equal number of factors as in the corresponding analysis on the WIA data.

## Results

Table 1 lists a number of characteristics of both the WIA and the WAO population. The largest differences between the two populations were observed in the age groups, with higher percentages in the oldest age groups of the WIA population. The differences between populations in gender, education and outcome of the assessment were much smaller.

**Table 1:** Characteristics of the population of claimants having a List of Functional Abilities in the CAMS computer system under two successive disability pension legislations.

Characteristics of the claimant	Disability pension legislation WIA/time period 2005-2007 (N = 50,931)	Disability pension legislation WAO/time period 2003-2005 (N = 80,968)
**Sex**:		
**Male**	47%	45%
**Female**	53%	55%
**Age group**:		
**< 25 years**	2%	5%
**25 - 35 years**	16%	21%
**35 - 45 years**	26%	28%
**45 - 55 years**	32%	31%
**>= 55 years**	24%	14%
**Educational level**:		
**primary education**	30%	27%
**lower secondary education**	33%	31%
**medium secondary education**	27%	30%
**higher or university education**	10%	12%
**The ultimate result of the assessment, i.e. the percentage reduction in earning capacity**:		
**< 35% reduction**	52%	52%
**35% - 80% reduction**	19%	19%
**> 80% reduction**	29%	29%

A factor analysis on the items of sections I and II of the LFA items produced eight factors with eigenvalues exceeding 1. However, the homogeneity of the content of the resulting factors was considered unsatisfactory, and a seven-factor solution produced results with greater homogeneity of content (figure 1). The reliabilities of the seven scales based on these factors are reported in the second column of table 2. Short descriptions of the items within each scale are listed in figure 1. The items of the first scale can be denoted as causes of work stress, for example, work demands, and stress-modifying characteristics. The second scale measures whether someone can perform independently in a work situation. 'Communication' is the title of the third scale, but the Cronbach's Alpha was only 0.54: clearly below the threshold and not acceptable. The common subject of the three items of the fourth scale was taking initiative: being able to start and execute a task without guidance. The common subject of the items of scale five was the interaction with other people as a requirement of the job. Cognitive functioning was the main subject of the sixth scale. The seventh scale related to an individual's goal-orientation. The Cronbach's Alpha of this scale, 0.59, nearly equalled the threshold value. Due to the common characteristics of the items within this last scale (the content), we nevertheless considered the results of this scale as just acceptable. The Cronbach's Alphas of the five scales above the threshold of the first two sections ranged from 0.73 to 0.86.

**Table 2:** The scales of the List of Functional Abilities under two successive disability pension legislations: reliability (Cronbach's Alpha) and 95% confidence interval.

Scale (number of items)	Disability pension legislation WIA/time period 2005-2007 (N = 50,931)	Disability pension legislation WAO/time period 2003-2005 (N = 80,968)
**Work stress (9)**	0.856 (0.854-0.858)	0.827 (0.826-0.829)
**No independence in performance (7)**	0.727 (0.723-0.731)	0.724 (0.721-0.727)
**Communication (6)**	0.537 (0.530-0.543)	0.491 (0.485-0.496)
**Taking initiative (3)**	0.749 (0.745-0.753)	0.718 (0.715-0.721)
**Social task demands (6)**	0.800 (0.797-0.802)	0.766 (0.763-0.768)
**Cognitive functioning (7)**	0.791 (0.788-0.793)	0.757 (0.754-0.760
**Acting efficiently (3)**	0.592 (0.586-0.598)	0.635 (0.631-0.640)
**Use of the legs (8)**	0.924 (0.923-0.925)	0.914 (0.913-0.915)
**Grip of the hand (5)**	0.937 (0.936-0.937)	0.903 (0.902-0.904)
**Use of the arms (7)**	0.865 (0.864-0.867)	0.843 (0.842-0.845)
**Posture of the trunk/back (3)**	0.720 (0.716-0.725)	0.730 (0.727-0.733)
**Use of the hand and fingers (7)**	0.762 (0.759-0.766)	0.683 (0.680-0.687)
**Use of the neck (2)**	0.691 (0.686-0.697)	0.675 (0.670-0.679)
**Movement of the trunk/back (4)**	0.612 (0.606-0.617)	0.583 (0.578-0.588)
**Working hours (2)**	0.973 (0.972-0.973)	0.968 (0.968-0.969)

**Figure 2:**
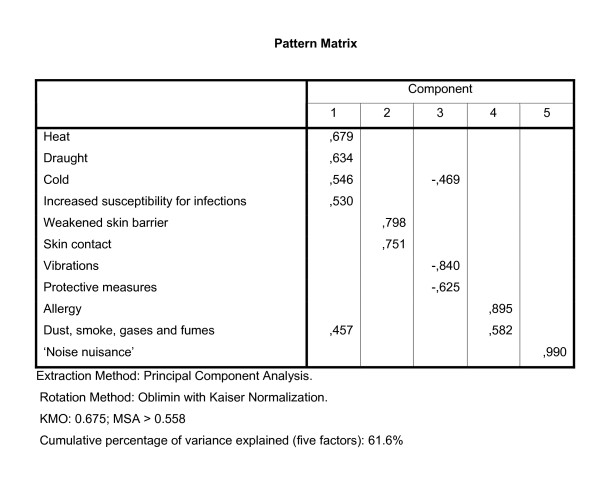
**Pattern matrix of the factor analysis on section III of the LFA, using WIA data**. Section III includes 11 items related to adjusting to the physical environment.

The analysis on the items of section III produced five factors (figure 2). The fifth factor would consist of only one item, 'noise', and therefore no scale could be based on this last factor. The Cronbach's Alphas of the other four scales ranged from 0.39 to 0.51, considerably lower than the threshold value of 0.60. We therefore decided not to use the scales of this section, and we did not repeat this analysis on the WAO data.

**Figure 3:**
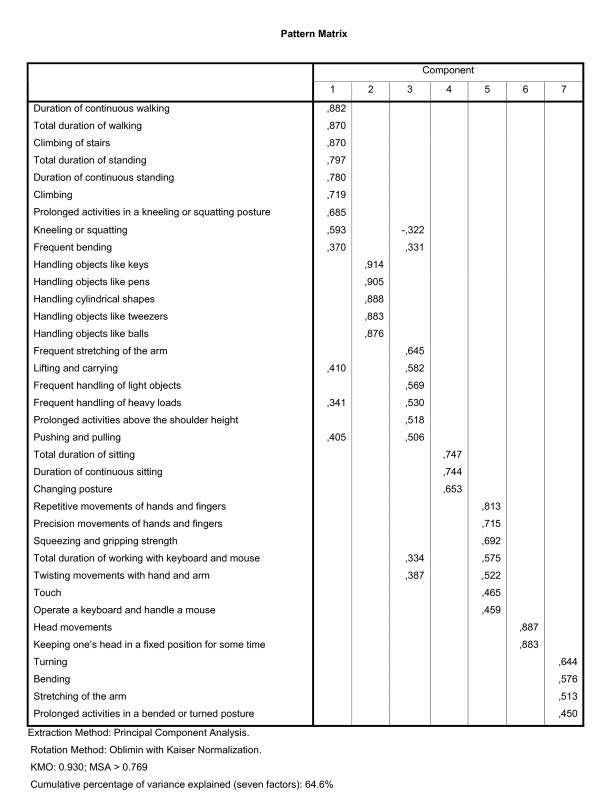
**Pattern matrix of the factor analysis on sections IV and V of the LFA, using WIA data**. Section IV includes 27 items on dynamic movements and section V includes 9 items on static postures.

Within sections IV and V of the LFA, a factor analysis on the WIA data identified seven factors (figure 3). The common denominator of the first scale was the use of the legs. The item 'frequent bending' had the highest coefficient on this scale. However, if one interprets 'frequent bending' as using the arms to manipulate something manually in a low position, for example to pick something up, then the item fits better in the scale 'Use of the arms'. Moreover, the coefficient of this item on the scale 'Use of the arms' was the second highest, and only slightly less than the highest one. We therefore decided to assign the item 'frequent bending' to the scale 'Use of the arms' (but not in the comparison with the dimensional structure within the WAO data, see below and table 3). The items of the second scale of this factor analysis assessed the ability to get a grip of objects of various shapes. The subject of the third scale was the use of the arms. The common denominator of the fourth scale was the sustained posture of the trunk. The fifth scale related to the use of the hands and the fingers, with the exception of the grip on various objects (which is measured by the second scale of this section). The sixth scale contained two items relating to the use of the neck, and the last scale was about the movements of the trunk (in a forced posture). The seven scales within these two sections had Cronbach's Alphas ranging from 0.61 to 0.94 (see table 2).

**Table 3:** The relation between the dimensional structures of the items of the sections IV^(a^^) ^and V^(b^^) ^of the LFA within the population of clients during the WIA legislation (rows) and the WAO legislation (columns).

	**Scale WAO: serial number of the scale**^**(c) **^**(number of items)**						
**Scale WIA (number of items)**	**1(8)**	**2(5)**	**3(7)**	**4(6)**	**5(2)**	**6(6)**	**7(2)**
**1. Use of the legs (9)**	8^(d)^			1			
**2. Grip of the hand (5)**		5					
**3. Use of the arms (6)**			6				
**4. Posture of the trunk/back (3)**				3			
**5. Use of the hand and fingers (7)**			1			6	
**6. Use of the neck (2)**					2		
**7. Movement of the trunk/back (4)**				2			2

(a) Section IV of the LFA: 27 items on dynamic movements.

(b) Section V of the LFA: 9 items on static postures.

(c) Scale WAO: serial number and title:

1. Use of the legs

2. Grip of the hand

3. Use of the arms

4. Bending or fixed posture of the trunk/back

5. Use of the neck

6. Use of the hand and fingers

7. Turning/reaching

(d) The numbers in the table denote the number of items that are allocated to a specific combination of a WIA scale and a WAO scale, for example 8 out of 9 items of the WIA scale 'Use of the legs' were placed in scale with the same name on the basis of the WAO analysis.

Considering the small number of items (three) of section VI, we did not use factor analysis for these items. The item 'maximum number of hours per day' correlated highly (r = 0.95) with the item 'maximum number of hours per week', but the correlation of each of these two items with the third item, i.e. 'day work or (type of) shift work', was considerably lower: just over 0.50. Moreover, addition of the third item decreased the Cronbach's Alpha of the two-item scale considerable: from 0.97 to 0.86. Therefore, a two-item solution was chosen.

**Figure 4:**
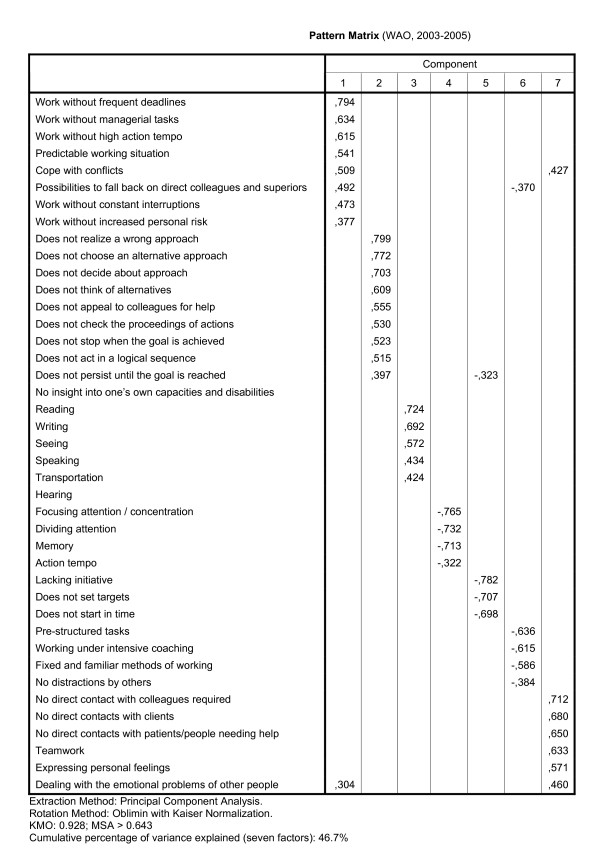
**Pattern matrix of the factor analysis on sections I and II of the LFA, using WAO data**.

**Figure 5:**
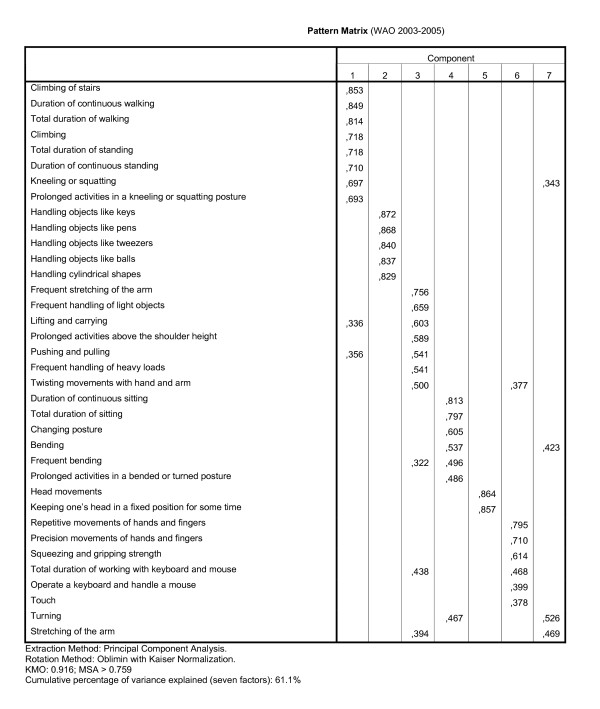
**Pattern matrix of the factor analysis on sections IV and V of the LFA, using WAO data**.

The stability of the results of the factor analyses on the combined sections I and II and the combined sections IV and V was studied by repeating the analyses for the WAO population. The pattern matrices of the factor analyses are presented in the figures 4 and 5, and the results of the reliability analyses in third column of table 2, in which the scale 'Working hours', based on section VI of the LFA, was added. The results of the factor analysis on the sections I and II for the WAO data are presented in figure 4. Differences between the dimensional structure of the WIA data and the WAO data are relevant if those would have led to an alternative distribution of the items over the scales. In table 4, the item allocation based on the WIA data (rows) was compared to that based on the WAO data (columns). Three differences in item allocations occurred, as can be seen in table 4, which also led to differences in the naming of the scales. The items of one WIA scale were split up into two WAO scales, and the items of two WIA scales were joined into one, and one item was assigned to another scale. For example, of the nine items of the scale 'Work stress' from the WIA analysis in the first row, eight items were placed in the first column/scale from the WAO analysis (also called 'Work stress'), and one in the sixth scale, called 'No interference during work'.

**Table 4:** The relation between the dimensional structures of the items of the sections I^(a) ^and II^(b) ^of the LFA within the population of clients during the WIA legislation (rows) and the WAO legislation (columns).

	**Scale WAO: serial number of the scale**^**(c) **^**(number of items)**						
**Scale WIA (number of items)**	**1(8)**	**2(10)**	**3(6)**	**4(4)**	**5(3)**	**6(4)**	**7(6)**
**1. Work stress (9)**	8^(d)^					1	
**2. No independence in performance (7)**		7					
**3. Communication (6)**			6				
**4. Taking initiative (3)**					3		
**5. Social task demands (6)**							6
**6. Cognitive functioning (7)**				4		3	
**7. Acting efficiently (3)**		3					

(a) Section I of the LFA: 26 items on personal functioning.

(b) Section II of the LFA: 15 items on social functioning.

(c) Scale WAO: serial number and title:

1. Work stress

2. No independent execution of tasks

3. Communication

4. Cognitive functioning

5. Taking initiative

6. No interference during work

7. Social task demands

(d) The numbers in the table denote the number of items that are allocated to a specific combination of a WIA scale and a WAO scale, for example 8 out of 9 items of the WIA scale 'Work stress' were placed in scale with the same name on the basis of the WAO analysis.

The pattern matrix of the factor analysis on the combined items of the sections IV and V of the LFA for the WAO data are presented in figure 5. In table 3, the item allocations based on the WIA data (rows) and on the WAO data (columns) are compared. Table 3 shows that four items switched from one WIA scale to another WAO scale.

The third column of table 2 shows the results of reliability analyses on the WAO data, using the scale compositions based on the WIA data. The Cronbach's Alpha of the scale 'Communication' was in both populations clearly below the threshold value of 0.60. The Cronbach's Alpha of the scale 'Acting efficiently' rose above the threshold value in the WAO population, whereas that of the scale 'Movement of the trunk/back' fell just below the threshold. Because of the small difference, this last Cronbach's Alpha was considerd acceptable.

## Discussion

The factor analyses on the WIA data within two groups of items, each group from two of the six sections of the LFA, produced 14 factors. Scale scores were based on these factors, and one extra scale was added, based on the items from section VI. Subsequent reliability analyses showed acceptable Cronbach's Alphas for 14 out of 15 scales, although the Alpha of one scale was just below the threshold we defined. The scales represented nameable concepts, and reduced the number of variables from 69 (including the three items from section VI, and not counting the 11 items from section III) to 15. The clustering of the items into scale variables produced no clear surprises, partly due to the separate factor analyses within (combined) sections. However, not all of the results on the WIA data could be replicated on the WAO data. The results of the factor analyses on the WAO data showed a different dimensional structure, and this result limits the possibilities for generalization of the dimensional structure of the LFA in the WIA data. Nevertheless, the reliability analyses on the WAO data, using the 14 WIA scales with an acceptable Cronbach's Alpha, showed that these scales kept acceptable or nearly acceptable Cronbach's Alphas.

Although the results of the factor analyses on the WAO data showed many similarities to those on the WIA data, the differences that were found were systematic, given the sizes of the two data sets. The most plausible cause for these differences is the one year vs. two year interval between the start of the sickness spell and the disability assessment. If these relatively minor difference produced a number of differences in scale composition, then even more and greater deviations may be expected if these analyses were repeated on data sets showing fewer similarities, for example originating from another country; recorded for another purpose or in another context; and so on. However, the scales that were developed on the WIA data can be used to describe subgroups of WIA claimants in the Netherlands. A provisional set of scales was used to produce profiles of subgroups according to diagnosis type [4]. To include a broad variety of relevant disabilities, the dimensions we found can be used as a starting point for a work disability assessment instrument, by selecting the relevant dimensions for the population concerned, and then selecting items within each of these dimension (not necessarily items of the LFA).

The LFA is intended to be a compact inventory of the functional abilities/disabilities of the claimant, as assessed by the IP. Although the items are classified into six sections, all separate items should be based on the assessment of a distinct ability. In other words, the absence or presence of a disability, and for about one-third of the items with an indication of the severity of the disability. The assessment of each disability aspect should be more or less independent of the assessments of other disabilities. However, some items of the LFA are, necessarily, mutually partly interdependent, mostly because they place a limit to two aspects of the same activity, of which the two highly correlated items of the scale 'working hours' form the most extreme example. Apart from these kind of (logical) interdependencies, relations between items will be based on characteristics of the population of claimants, such as: the prevalence of a disease with a typical pattern of disabilities; the co-morbidity of certain diseases; more than one disorder leading to the same pattern of disabilities; and so on. Because the items of the LFA were not selected to measure a common dimension, we regarded a Cronbach's Alpha of 0.60 as acceptable, which is lower than in many other studies. From this perspective, the reliabilities of some of the scales we identified in the results section may be only moderate in comparison with reliabilities found in other studies, but can be regarded as substantial in the present context, with the exception of the scale 'communication'.

The Cronbach's Alphas of 14 scales (the scale 'Communication' excluded) were acceptable (or nearly acceptable) in the WIA population, and this remained so in the WAO population. These 14 scales can be applied for a compact description of the nature of the disabilities of claimants in the WIA population.

The number of scales we identified, 15 scales (including the scale 'Communication'), was more than twice the number of scales Brage and colleagues [12] discerned in their study of the Norwegian Function Assessment Scale, which totalled 7 scales. The Norwegian study identified four factors/scales within the physical domain, three of which show resemblance to scales we identified: 'Walking/standing' ('Use of the legs'); 'Lifting/carrying' ('Use of the arms'); and 'Sitting' ('Posture of the trunk/back'). The Norwegian scale 'Holding/picking up things' seems to combine elements from our scales 'Grip of the hand' and 'Use of hands and fingers'. The last two physical scales we identified, i.e. 'Use of the neck' and 'Movement of the trunk/back', were not found in the Norwegian study.

The above-mentioned Norwegian study identified three mental factors/scales. The last scale, 'Senses', was composed of the items 'watching television' and 'listening to the radio'. These items show the most resemblance to those in the scale 'Communication' of the LFA. The reliability of the scale 'Senses' was relatively low compared to the other scales within the Norwegian study [12], but considerably higher (0.76) than that of our scale 'Communication'. The remaining two scales do not bear a clear resemblance to any of the scales we found in the present study. The differences in the number and the nature of the dimensions can presumably ascribed in part to the main differences between the studies in the input of the analysis: professional assessment registrations vs. self-evaluation questionnaires; 80 vs. 39 items; four separate analyses on subgroups of items vs. one analysis on all the items; a sick leave duration of two years vs. six weeks; and differences in the context/content of the items. These last differences in context/content may be related to the observed lack of resemblance, particularly in the mental dimensions, although both lists are partly based on the ICF. In the Norwegian questionnaire, the emphasis is on limitations in everyday actions and activities, whereas many of the items of the LFA relate disability to work situations. This may influence the subject matter of the items, and the thresholds for dysfunction/disability. The observed differences make clear that trying to generalise the dimensional structure within the functional abilities of work disabled over countries is not a realistic target at the moment with the present differences, for example, in legislation, in measuring instruments, and so on. The identification of large groups of workers with similar disabilities in different countries seems to be more feasible. Similar disabilities may require similar low-demand jobs to employ these individuals. Identifying the similarities in the disabilities and, subsequently, in the corresponding low-demand jobs would make the results of these types of studies applicable in more than one country. The low-demand jobs selected for specific groups of disabled within one country, can be used to identify low-demand jobs for similar disabled people in other countries.

The EUMASS working group for the ICF proposed an ICF core set for functional assessment in disability claims of 20 categories [14]. Compared to our set of 15 scales, some categories of the ICF core set are more general than our dimensions, while other categories are more detailed, on the level of items of the LFA. For example, the category 'Handling stress and other psychological demands' is more general than the dimensions we found within the sections I and II of the LFA, and other categories equal almost all items of the scale 'Communication' of the LFA. The category 'Sensation of pain' of the ICF core set has no equivalent item in the LFA, and the subjects in section III of the LFA about the physical environment are not addressed in the ICF core set. The differences between the two sets can probably be ascribed in large part to the differences in goals of the two studies/development projects.

The data of our study were collected in regular disability assessments of IPs as part of the public work disability insurance system of employees in the Netherlands. The data for all claimants were registered in LFA files, with the exception of two types of claimants, the first of which was regarded as hardly disabled, and the other as too severely disabled (see the method section). The data for all other claimants in the two intervals of data collection were included. Therefore, the population of our study was highly diverse and substantial in size, and those characteristics were major strengths of this study. In addition, the earlier-described context for the data collection was beneficial to the relevance and the quality of the items of the LFA as indicators of the work capacity of the claimants. However, the LFA was developed and the data were registered for the purpose of the assessment of work disability, and although it was based in part on the ICF, the comparability of the LFA items with items of other well-known instruments was only of minor importance in the development of the LFA. This limits the possibilities to generalise the results to other countries.

Another disadvantage of the use of existing LFA data was that we did not monitor the quality of the assessments (and the registration of the assessments). However, various estimates on the reliability of the LFA and related data were produced in four other studies we will discuss below.

Spanjer et al. [17] reported the inter-rater reliability of the items on physical abilities of the LFA in a study of a new method for the assessment of work disability. In this study, 62 work disability claimants were interviewed and examined by two IPs independently. The authors reported a reasonable to good inter-rater reliability of the items, for both the newly introduced method and the usual method of assessment. The latter was also applied in our study. In an earlier study, Spanjer et al. [18] reported a comparable inter-rater agreement of IPs on the items on physical abilities of the LFA in simulated assessments of claimants with low back pain or a lower extremity disorder, based on written interview reports of assessments in practice. Before that, similar results were found in a study of Spanjer [19] into the predecessor of the LFA, the work capacity profile of the Function Information System (FIS). The items of this predecessor of the LFA resemble the items of the present LFA, and these results are therefore relevant. The assessments of the IPs in this last study [19] were based on video recordings of interviews of other IPs with claimants.

Brouwer et al. [20] studied the applicability of the items on physical abilities of the LFA in the work-related functional status assessment of chronic low back pain patients in a rehabilitation centre. The reliabilities of the items on physical abilities of the LFA were judged as generally insufficient for that purpose, as were those of the aforementioned predecessor, the work capacity profile of the FIS. Brouwer et al. [20] restricted their negative conclusions about the LFA to the application in the rehabilitation domain. Possibly, some patients present their health problems differently to the physician, as compared to disability benefit claimants [21,22]. Therefore, their study is less relevant for the quality of the LFA in disability assessments than the studies of Spanjer and colleagues.

The characterisation of subgroups of disabled employees by the scale variables can be used to identify relatively accessible jobs for that group. In other words, jobs that are low in the corresponding work demands, and with hardly any other high-level demands and job requirements that might form an impediment for many employees to work in those jobs. Subsequently, these jobs can be used in work rehabilitation and work counselling for disabled employees with a specific type of disability. In addition, in occupational health and in social security, professionals can utilise these jobs to illustrate the existence of the residual work capacity of sick-listed employees in existing regular jobs. Without further research, our scale variables as such can only be applied in research in the context of the Dutch social security system, for example: to study the broad effects of a rehabilitation programme for disabled employees; to monitor the functional abilities in the population of claimants; to give a concise description of subgroups of disabled employees; and so on.

## Conclusions

The interrelations between the items of the LFA were dependent of details of the legislation and/or the composition of the population. Thus, the results of this study cannot be fully generalised to other situations, due to the differences in legislation, assessment regulations, instruments, population, and so on. However, the scales we constructed may be applied to construct a concise description of the functional abilities of groups of claimants in the Netherlands, for example, classified according to age class or diagnosis type. The results of this study may further be useful for the assessment of the relevant functional abilities and disabilities of large groups of long-term sick-listed employees to identify the impediments to their work resumption in their former job, as well as in various other jobs. In addition, the corresponding work demands can be identified, and jobs low on those demands can be selected as being most accessible for the specific type of disabled employees, particularly severely disabled employees. These low-demand jobs could be utilised in rehabilitation efforts in the Netherlands, and, under the assumption of comparable subgroups of disabled employees, also in other countries.

## Abbreviations

CAMS: Claim Assessment and Monitoring System; C.I.: confidence interval; EUMASS: European Union of Medicine in Assurance and Social Security; FIS: Function Information System; ICF: International Classification of Functioning, Disability and Health; IP: insurance physician; LE: labour expert; LFA: List of Functional Abilities; NFAS: Norwegian Function Assessment Scale; OECD: Organisation for Economic Co-operation and Development; WAO: the former Dutch law on work disability, the predecessor of the WIA; WIA: the present Dutch law on work disability

## Competing interests

The authors declare that they have no competing interests.

## Authors' contributions

JPJB wrote this manuscript. HPGM, AJMS and AJvdB advised on the methods used, and commented on the manuscript. All authors have read and approved the final version of this manuscript.

## Pre-publication history

The pre-publication history for this paper can be accessed here:

http://www.biomedcentral.com/1471-2458/11/99/prepub
